# Influence of bio-fertilizer containing beneficial fungi and rhizospheric bacteria on health promoting compounds and antioxidant activity of *Spinacia oleracea* L.

**DOI:** 10.1186/s40529-017-0189-3

**Published:** 2017-08-16

**Authors:** Muhammad Khalid, Danial Hassani, Muhammad Bilal, Fayaz Asad, Danfeng Huang

**Affiliations:** 1 School of Agriculture and Biology, Shanghai Jiao Tong University, Shanghai, 200240 People’s Republic of China; 2 State Key Laboratory of Microbial Metabolism, School of Life Sciences and Biotechnology, Shanghai Jiao Tong University, Shanghai, 200240 China; 3 Key Laboratory of Alpine Ecology and Biodiversity, Key Laboratory of Tibetan Environment, Changes and Land Surface Processes, Institute of Tibetan Plateau Research, Chinese Academy of Sciences, Beijing, 100101 China

**Keywords:** Bio fertilizer, Arbuscular mycorrhizal fungi, Rhizospheric bacteria, Health-promoting compounds, Spinach

## Abstract

**Background:**

This study evaluates the influences of bio fertilizers containing mycorrhizal fungi (*Glomus fasciculatum, Glomus mosseae*) individually or in combination with N-fixer (*Azotobacter chroococcum*), K solubilizer (*Bacillus mucilaginous*) and P solubilizer (*Bacillus megaterium*) on soil fertility and phytochemical levels of spinach.

**Results:**

Root colonization by mycorrhizal fungi was increased in the presence of bacterial inoculation in comparison to individual inoculation treatments. Inoculation of bio fertilizer containing mycorrhizal fungi and bacterial species considerably augmented the concentration of total phenolic compounds, flavonoids and phenolic acid contents. The 1, 1-diphenyl-2-picrylhydrazyl (DPPH) scavenging capacity of spinach was found to be positively coincided with flavonoid contents, while partially correlated with total phenolic compounds and phenolic acids. Further, the HPLC analysis showed that significantly higher antioxidant activity of spinach was correlated with quercetin contents and chlorogenic acid. Chlorophyll contents were also increased following the bio fertilization treatments.

**Conclusion:**

Results revealed that these microbes are useful tool for improving health promoting compounds in spinach.

## Introduction

The consumption of fruits and vegetables could increase the human innate immunity against chronic diseases (Bagchi et al. 2003; Yochum et al. 1999). The phytoconstituents including polyphenols, quercetin and flavonoids are largely demonstrated as important antioxidants and exhibit profound radical scavenging capabilities (Bravo 1998; Chu et al. 2000; Duthie et al. 2000; Gil et al. 1999; Middleton and Kandaswami 1994). *Spinacia oleracea* L. is one of the most important and commonly consumed leafy vegetable. It is commercially known as spinach which is claimed to possess therapeutic properties and being a rich source of flavonoids as well as phenolic compounds besides its economical and ease of availability (Bunea et al. 2008; Ferreres et al. 1997; Metha and Belemkar 2014; Sultana and Anwar 2008). The pro-health properties of spinach are attributed to its low calorific value, and its large supply of vitamins, micro- and macronutrients and others phytochemicals, including polyphenols and fiber (Llorach et al. 2008). The quality of fresh vegetables could be assessed based on their nutritional value, growing conditions and usage of fertilizer. Despite the fact that the genetic modification and agronomic manipulation methods are widely used to improve the nutritional value of plants, the inadequate public acceptance and soil specificity of genetically modified food are still the challenges (Martínez-Ballesta et al. 2008).

Started about 60 years ago, several studies have revealed the potentiality of beneficial microbes in increasing the plants resistance to biotic and abiotic stresses through the up-production of secondary metabolites (Shen 1997). Beneficial bacteria or fungi inhabit various sites such as plants rhizosphere, while others colonize on rhizoplane or even intercellular spaces (McCully 2001). Former studies revealed that phosphate and potassium solubilizing bacteria decompose the phosphate and potassium from their sources and make them available to the plants, assisting essential mineral uptake. Plant growth promoting rhizobacteria (PGPR) thrives in the rhizosphere of plants. It is worth mentioning that a substantial number of bacterial and fungal species entertain a functional relationship and establish an integrated system with the plants. They enhance the plant growth either by assisting in essential nutrients acquisition (minerals, nitrogen and phosphorus), eliciting pertinent hormones or acting as bio-control agents to reduce the inhibitory effects of various pathogens (Yang et al. 2009). Some strains such as *Azotobacter chroococcum* and *Azospirillum brasilense* have shown to possess properties of biological nitrogen fixation both in legume and non-legume, exerting a positive effect on overall physiology and development of the plants (Dobbelaere et al. 2001; Goldstein and Liu 1987). Former literature survey revealed 30% improvement in the yield of wheat by the *Azotobacter* inoculation (Zablotowicz et al. 1991). Likewise, root/shoot length and dry weight has been significantly increased in tomato, lettuce and canola by inoculation of *Pseudomonas putida* and *Pseudomonas fluorescens* (Glick et al. 1997; Hall et al. 1996).

Arbuscular mycorrhizal fungi (AMF) are associated with majority of the plants, growing under natural conditions and its contribution for micronutrients uptake is well-documented in the previous reports. Moreover, these beneficial microbes protect the plants from oxidative stress by synthesizing antioxidant enzymes including, peroxidase, catalase, superoxide and non-enzymatic antioxidants such as glutathione, ascorbate and α-tocopherol; hence, providing an suitable way to replace the hazardous agricultural chemical and agro-ecosystems destabilizing fertilizers.

The current study was appraised to evaluate the influence of beneficial bacteria (*A. chroococcum, Bacillus megaterium* and *Bacillus mucilaginous*) and fungi (*Glomus fasciculatum* and *Glomus mosseae*) on the antioxidant properties and physiological activities of *S. oleracea* L. and to develop an alternative method for improving the quality of health promoting phytochemicals and anti-radical activity of spinach.

## Materials and methods

### Chemicals and reagents

Standard laboratory grade chemicals/reagents including, Folin–Ciocalteau reagent, 2,2-diphenyl-1-picrylhydrazyl radical (DPPH), gallic acid, ascorbic acid, Dithiothreitol (DTT), and (±)-6-Hydroxy-2, 5, 7, 8-tetramethylchromane-2-carboxylic acid (Trolox) were mainly procured from Sigma-Aldrich (USA) and DSL Chemicals (Shanghai) Co., Ltd. All the experimental works were carried out in the Joint Laboratory of Digital Horticulture, School of Agriculture and Biology, Shanghai Jiao Tong University, Shanghai China.

### Soil

Soil sample, was collected from the botanical garden of School of agriculture and biology, Shanghai Jiao Tong University, China. The collected soil was air-dried, grinded, passed through a sieve (2 mm for chemical analysis and 8 mm for pot experiment) and mixed thoroughly. Soil was autoclaved three times and analyses were made prior to seeding. Basic properties of soil were; pH, 7.32; EC, 0.14 (dS/m); available N, 111.6 (ppm); available P, 181.7 (ppm), available K, 306.8 (ppm), cation exchange capacity (CEC), 13.2 (cmol(+)/kg); NH_4_^+^, 7.86 (ppm); NO_3_^−^, 2.67 (ppm); total C, 1.92 (%); total N, 0.19 (%); total K, 2063 (ppm).

### Inocula development and bio-fertilizer

Sterilized peat moss was chosen as a carrier for the rhizobacteria including *A. chroococcum* (nitrogen fixer), *B. megaterium* (phosphate solubilizer) and *B. mucilaginous* (potassium solubilizer) inoculums. All the bacterial strains were cultured in Luria–bertani (LB) broth at 28 °C for 48 h in a rotary shaker at an agitation speed of 120 rpm. After designated time period, the culture density was measured by means of a haemocytometer (improved neubauer counting chamber) following the previously described method (Wu et al. 2005). The strains were centrifuged at 5000 rpm (at 4 °C) and resulting cells were thoroughly mixed with the sterilized peat moss. Mixture acting as the microbial inoculum contained a final population density of 1.33 × 10^8^, 2.08 × 10^8^, 2.66 × 10^8^ cfu g^−1^ inoculum (wet weight) for K, P and N fixing bacteria, respectively. The sand-based two fungal inoculums consisting hyphae and spores were purchased from the Central bureau voor Schimmel cultures, Fungal Biodiversity Centre, Institute of the Royal Netherlands Academy of Arts and Sciences (KNAW).

### Experimental set up

Greenhouse trial was conducted to investigate the efficiency of different combination of beneficial fungi and rhizobacteria on the growth and antioxidant profile of spinach. According to the treatment design, 15 g of sand-based mycorrhizal inoculum and 2 g of peat moss-based bacterial inoculum were inoculated into each pot according to treatment design. Treatments were set up for comparison, where 1st treatment (T1) considered as control with no fertilization, while the 2nd (T2) and 3rd (T3) treatment contained chemical fertilizers (KCl, KH_2_PO_4_ and urea) and autoclaved organic bio-fertilizer, respectively. The detailed information of experimental design has been listed in Table 1.

**Table 1:** Experimental design

Treatments	T1	T2	T3	T4	T5	T6	T7
Bacteria inoculation	No	No	No	Yes	No	No	Yes
Mycorrhizal fungi	None	None	None	None	Yes	Yes	Yes
Fertilizer used	None	CF	OF^A^	None	None	None	None
Code	Control	CF	OF	B	GF	GM	B + GF + GM

*CF* chemical fertilizer, *OF* ^*A*^ organic fertilizer (autoclaved bio-fertilizer), *B Azotobacter chroococcum* + *Bacillus megaterium* + *Bacillus mucilaginous*, *GF Glomus fasciculatum*, *GM Glomus mosseae*

### Seeds germination and analysis

The seeds of spinach were procured from Shanghai WELLS seed Co., LTD. Prior to sowing, the seeds were surface disinfected three times by soaking in 70% ethanol for 5 min and then in distilled water. After seed germination in each pot (height, 10 cm; bottom diameter 9 cm, top diameter 10 cm, soil, 800 g per each pot), the seedling were thinned and only two seedling per pot were allowed to continue their growth. The pots were placed randomly in a greenhouse with an average temperature of 21 ± 5.0 °C and watered (twice a week) with distilled water to maintain the appropriate soil humidity level. After 45 days, the plants were harvested, collected and used for analytical purposes. Each treatment was carried out in 15 replicate pots to maintain the reproducibility of the data.

### Fungi and bacteria colonization assay

At harvesting, the root were washed, treated with KOH (10.0%) for 20 min at 90 °C, acidified with HCL 1.0% for 3 min and then stained with trypan blue 0.05%, and subjected to fungal root analysis in a manner as described previously (Giovannetti and Mosse 1980). Differentiating media with suspension dilution techniques from the soil samples were used to isolate and measure bacteria growth. For phosphate solubilizing bacteria:, NaCl 0.4 g, (NH_4_)_2_SO_4_ 0.6 g, Ca_3_(PO_4_)_3_ 9.0 g, KCl 0.3 g, FeSO_4_·7H_2_O 0.03 g, MgSO_4_·7H_2_O 0.5 g, MnSO^4^·4H_2_O 0.03 g, agar 21.0 g, glucose 10.0 g, sterilized water 1.0 L, pH 7.0; for Nitrogen fixer:, NaCl 0.3 g, 2% congo red solution 6 mL, K_2_HPO^4^ 0.4 g, MgSO^4^·7H_2_O 0.2 g, 3 drops of 1% FeCl_3_ and 1% MnCl_2_ solution, agar 20.0 g, glucose 10.0 g, sterilized water 1.0 L, pH 7.0; for potassium solubilizing bacteria: Na_2_HPO_4_ 2.0 g, FeCl_3_ 0.005 g, CaCO_3_ 0.1 g, MgSO_4_· 7H_2_O 0.5 g, glass powder 1.0 g, agar 20.0 g, sucrose 5.0 g, 1.0 L distilled water, pH 7.0 (Wu et al. 2005).

### Extract preparation of health-promoting compounds

The phenolic compounds were extracted following the method previously described (Khalid et al. 2017). The phonolic compounds were extracted following the method previously described Frozen leaf tissue (2.5 g) was grinded with a mortar and pestle using 15 mL of 50% (v/v) acidified methanol (0.1 M HCl) and the phenolic compounds were extracted for 20 min at room temperature, then centrifuged at 9000g for 30 min. This procedure was repeated three times and the supernatants were combined to produce a crude extract of polyphenols. The raw methanolic extract was then evaporated to dryness under a vacuum at a temperature of 40 °C and rinsed with 100% methanol to a final volume of 10 mL.

### Study of health promoting phytochemical

#### HPLC–MS analysis

Quantitative assessment of phenolic compounds was carried out through HPLC–MS (LTQ XL, Thermo Fisher Scientific, San Jose, CA, USA) analysis (Świeca et al. 2012). The HPLC–MS system was equipped with a ternary pump, auto sampler, and thermostatic column compartment, diode array detector (Surveyor, Thermo Fisher), and a linear ion trap mass spectrometer (LTQ XL, Thermo Fisher Scientific, San Jose, CA, USA) equipped with an electrospray ionization (ESI) source. A CORTECS C18 column (2.1 mm × 100 mm, 2.6 µm; Waters) was used; the column temperature was maintained at 35 °C. The mobile phase A (0.1% formic acid/water) and B (acetonitrile) was used, the gradient program was as follows: 0–2 min 5.0% B; 4–11 min 15–35% B; 15–17 min, 100% B; 17.5–22 min, 5.0% B; flow rate was 0.25 mL min^−1^, the injection volume was 4 μL. UV detection was performed at 270 and 370 nm, the wavelength was scanned from 200–600 nm. MS was scanned in ESI source in negative mode, mass range: *m/z* 92–1000; source voltage was 3.5 kV, capillary temperature was 350 °C, sheath gas flow was 35, aux gas flow was 15.0, sweep gas flow was 1.0, and capillary voltage was 43 V. Data acquisition, handling, and instrument control were performed using Xcalibur 2.3.1 software.

### Determination of total phenolic contents, flavonoids and phenolic acid

Total phenolic contents (TPCs) were determined by the method as reported earlier (Singleton et al. 1999). Briefly, 0.5 mL H_2_O in combination with 2.0 mL Folin–Ciocalteau reagent (1:5 H_2_O) was mixed with 0.5 mL of the plant sample. After 3–5 min of incubation at room temperature, 10 mL of Na_2_CO_3_ (10%, *w/v*) was added to the mixture and incubated at room temperature for 30 min. Optical density of each sample was recorded at 725 nm in a UV–Vis spectrophotometer (HITACHI, U-2900) using Gallic acid as standard.

Total flavonoid contents (TFCs) were estimated in a manner described by (Lamaison and Carnat 1990). Shortly, 1.0 mL sample extract was allowed to react with 1.0 mL of aluminium chloride (AlCl_3_·6H_2_O) solution (2.0%, w/v) for 10 min at room temperature and absorbance was monitored at 430 nm.

A previously reported method of (Szaufer-Hajdrych 2004) was followed for measuring the phenolic acid contents (PACs) in the extracted sample. To this end, 1.0 mL of sample was thoroughly mixed with a combination of 5.0 mL of distilled water, 1.0 mL HCl (0.5 M), Arnov reagent and NaOH (1 M) followed by OD measurement at 490 nm.

### Chlorophyll measurement

Chlorophyll content was evaluated as reported (Lin et al. 2013). Freeze-dried leaves samples were grinded with acetone, centrifuged at 13,000 rpm for 5.0 min. Supernatants were collected and spectrophotometrically measured at 663 and 645 nm to analyze chlorophyll a, and chlorophyll b.

### Total antioxidant activity

For antioxidant activity, free radical scavenging assay was carried out using 1, 1-diphenyl-2-picrylhydrazyl (DPPH) as described (Brand-Williams et al. 1995; Khanam et al. 2012). An 80 µL of methanolic extract was mixed with 1.92 mL DPPH solution, and absorbance was immediately noted at 515 nm. The quenching affinity of the sample was measured according to the relation given below.

$${\text{Scavenging }}\left( \% \right) \, = \, \left[ {\left( {{\text{AC}} - {\text{AA}}} \right)/{\text{AC}}} \right)] \, \times 100$$

where, AC represents the control absorbance at “0” min and AA denotes the absorbance of sample after “*t*” time.

### Statistical analysis of data

All the analytic determinations were carried out in triplicates. Statistical analysis was performed using STATISTICA 7.0 software for mean comparison using Tukey’s test at the significance level of *P* < 0.05.

## Results and discussion

**Fig. 1:**
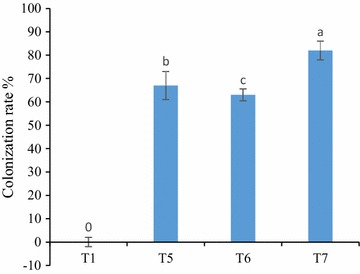
Arbuscular mycorrhizal fungi infection rate of plant root after harvest in co-inoculated plants

Colonization assay results in Fig. 1 manifest that the plant roots were found to be significantly colonized by Arbuscular mycorrhizal fungi (AMF) as compared to un-inoculated control treatments. Inoculation with beneficial bacteria and both *G. fasciculatum* and *G. mosseae* in T7 treatment led to augmented root as compared to T5 and T6. It has been demonstrated that the rhizobacteria can elicit the ability of mycorrhizal fungi in colonizing plant roots (Fitter and Garbaye 1994). Though, the exact mechanism underlying this triggering pathway is still unclear, but the up-production biosynthesis of plants hormones and vitamins as a result of bacterial infection may be involved in this process (Barea et al. 2002). The presence of beneficial rhizobacteria might cause the germination of a large number of spores by fungi, and as a consequence higher chances to infect/colonize plant roots (Tandon and Prakash 1998). It is not surprising that some beneficial bacteria actively secrete pectinolytic and cellulolytic enzymes that could further assist in mycorrhizal infection (Verma et al. 2001). In an earlier study, *A. chroococcum* has been isolated from the rhizospheric soil of *Spinacia oleracea* (Jiménez et al. 2011). Similarly, (Çakmakçı et al. 2007) reported that *B. megaterium* and *B. mucilaginous* successfully colonized rhizospheric zone in wheat and spinach. Recently (Song et al. 2015) observed a great synergy between the above mentioned strains and their aptitude to improve the quality of soil.

The present findings indicate that the level of bacteria *(A. chroococcum, B. megaterium, B. mucilaginous*) in the rhizosphere as well as AMF integration in the roots was different in co-inoculated treatments (Fig. 1; Table 2). The numbers of bacteria and AMF infection were found to be higher in T7 compared to T4, 5 and 6 treatments (Tables 1, 2) suggesting the auxiliary property of micro-organism in combination with each other.

**Table 2:** Population of beneficial bacteria in the rhizosphere of co-inoculated spinach after 45 days of growth

Treatment	NFB (10^4^ cfu/g dry soil)	PSB (10^6^ cfu/g dry soil)	KSB (10^6^ cfu/g dry soil)
T4	30.6 ± 3.30 b	44.1 ± 0.09 b	44.2 ± 1.50 b
T7	63.1 ± 11.5 a	79.8 ± 3.55 a	74.7 ± 3.91 a

*NFB* nitrogen fixing bacteria, *PSB* phosphate solubilizing bacteria, *KSB* potassium solubilizing bacteria

### HPLC analysis results from spinach extract

The possible compounds were identified by MS1, MS2 fragments and compared with the reported literatures (Kim et al. 2008; Ribas-Agustí et al. 2011; Złotek et al. 2014). The retention time, m/z in negative mode, MS2 fragments and the possible chemical name are listed in Table 1. The major antioxidant and health benefiting compounds that were identified such as caffeic acid, ferulic acid, flavones (luteolin), flavonols (quercetin, kaempferol), isorhamnetin-3-gentiobioside-7-glucoside have been reported variously in previous studies in spinach (Alarcón-Flores et al. 2014; Justesen 2001; Nuutila et al. 2002). Chlorogenic acid, coumaric acid are also reported by Okazaki and coworkers while analyzing the effect of nitrogen concentration on the constituent’s profile of spinach (Okazaki et al. 2008).

### Influence of experimental treatments on health promoting compound in spinach leaves

**Fig. 2:**
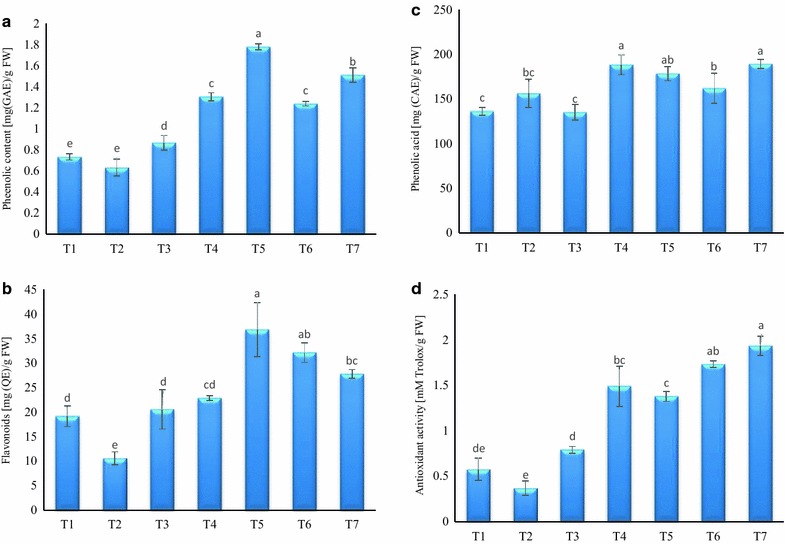
Influence of selected treatments on **a** phenolic contents, **b** flavonoids, **c** phenolic acids, **d** antioxidant activity

In view of potential medicinal and preventive roles of secondary metabolites in chronic diseases such as cancer, cardiovascular and neurodegenerative disorders (Riedel et al. 2012), the health promoting metabolites of spinach were analyzed. Results showed that the total phenolic, flavonoids and phenolic acid contents were found to be significantly varied under different experimental treatments. The amount of total phenolic contents was 58.72 and 51.43% higher in T5 and T7 treatment, respectively, as compared to un-inoculated control plants (Fig. 2). Similar to our findings, the eliciting effect of *A. chroococcum* and *G. fasciculatum* on various other plants growth and total phenolic compounds have been observed (Baslam et al. 2011; Teixeira da Silva and Egamberdieva 2013). The flavonoid content was evaluated under different treatments and results are shown in Fig. 2. It was observed that experimental plants co-inoculated with *G. fasciculatum* and *G. mosseae* showed the highest content of flavonoids in T5 and T6, which were 48.02 and 40.46% greater than that of control plants. Investigation of antioxidant biosynthesis and induction of phytochemicals including flavonoids by AMF has earlier been documented in several reports (Carlsen et al. 2008; Eftekhari et al. 2012; Nisha and RajeshKumar 2010). Similarly, phenolic acids were also recorded to be significantly higher in all treatments compared with control. Nevertheless, the T4 treatment and T7 treatment led to the 27.68 and 28.07% higher values, respectively in comparison to un-inoculated plants. The results were in good agreement with (Taie et al. 2008) who have reported up to 75% improvement of phenolic acid biosynthesis in Rhizobacteria inoculated soybean seedling.

### Antioxidant activity

The results of antioxidant activity analyzed through DPPH scavenging assay are portrayed in Fig. 2. It was observed that utmost antioxidant activity was determined in T7 (1.9 mM Trolox/g FW) elicited by bacterial and fungal combination followed by T6 (1.8 mM Trolox/g FW) treated with fungus *G. mosseae*. Observed improvements in antioxidant level were 70.17, 66.66 and 61.29% for T7, T6 and T4, respectively, as compared to control (without any bacterial and fungal inoculation). The DPPH scavenging capacity of spinach was found to be positively coincided with flavonoid contents, while partially correlated with total phenolic compounds and phenolic acids. Polyphenols are important class of biologically-active compounds with extensively reported antioxidant characteristics. However, other properties, such as, the capability to suppress enzymes [lipoxygenase or cyclooxygenase (COX)] involved in the inflammation process have recently taken more attention (Gawlik-Dziki et al. 2011; Mulabagal et al. 2010). Nevertheless, earlier reports highlighted that antioxidant capacity of any plant extract highly depends on the type and relative proportion of phenolics presence. In our study, the significantly higher DPPH scavenging potential of spinach might be positively correlated with quercetin contents and chlorogenic acid (Table 4). The results strongly corroborates with (Kim et al. 2007; Liu et al. 2007) who observed pronounced antioxidant activity of lettuce in the presence of quercetin and chlorogenic acid, while luteolin and caffeic acid negatively influences the antioxidant activity. In addition to phenolic compounds, several other bioactive constituents such as carotenoids and vitamins particularly vitamin C potentially contribute a key role in the elicitation of antioxidant potentialities of the plants (Sun et al. 2012).

### Chlorophyll content

Green leafy vegetables are known to be the richest source of major dietary metabolites. Spinach possess a considerable amount of chlorophyll which play critical role in reducing the risk of cancer and cardiovascular diseases (CVDs) (Caldwell and Britz 2006). It has been demonstrated that antioxidant rich diets, particularly chlorophyll inhibit the functions of COX-1 and COX-2 (Kim et al. 2007; Mulabagal et al. 2010). There are a variety of previously reported studies concerning the stimulating effect of rhizobacteria combination in co-cultivation with *Ociumum basilicm* L. (Vivas et al. 2003). The responses of chlorophyll contents in spinach treated with different elicitors are illustrated in Table 4. Chlorophyll a content was recorded to be significantly higher in plants co-inoculated by *A. chroococcum*, *B. megaterium* and *B. mucilaginous* bacterial strains (T4) in contrast with the control. Whereas, no significant difference was observed in chlorophyll a content between the control and T3, T5 and T6 treatments (Table 4). The co-cultivation of plants with Mycorrhizal fungi as well as chemical and organic fertilizer had no important impact on chlorophyll b content whereas a significantly higher chl b content was recorded in experimental treatment inoculated with bacterial strains (T4) (Table 3). Bacterial inoculation had also an elevating role in total chlorophyll content (a + b). Chemical fertilizer (T2) as well as bacterial and fungal strains combination treatment (T7) displayed higher values 458.66 ± 7.27 b and 515.79 ± 13.15 c, respectively. No significant difference was observed in other treatments (Table 4).

**Table 3:** List of constituents based on the of HPLC analysis of *Spinacia oleracea* L extract

*t*_*R*_	Negative mode (*m*/*z*)	Possible compound	Chemical name	MS^2^ (−)
0.93	193	C_10_H_10_O_4_	Ferulic acid	175
0.98	179	C_9_H_8_O_4_	Caffeic acid	161, 143
1.58	353	C_16_H_18_O_9_	Chlorogenic acid	335, 249
1.67	285	C_15_H_10_O_6_	Kaempferol	267, 225
4.26	285	C_15_H_10_O_6_	Luteolin	267, 225, 153
6.85	295	C_13_H_12_O_8_	Caffeoylmalic acid	163, 149
7.10	163	C_9_H_8_O_3_	Coumaric acid	119
7.60	787	C_33_H_40_O_22_	Quercetin-3-gentiobioside-7-glucoside	772, 769, 655, 637,505, 373, 330, 313
8.20	801	C_34_H_42_O_22_	Isorhamnetin-3-Gentiobioside-7-glucoside	786,769, 669, 651, 387, 345, 330, 329
8.51	465	C_20_H_22_O_9_	Cassiaside	437, 407, 379, 259, 241
8.63	301	C_15_H_10_O_7_	Quercetin	283, 265 255, 237
8.96	337	C_16_H_18_O_8_	5-*p*-coumaroylquinic acid	277, 191
10.3	521	C_30_H_34_O_8_	Benzoylgomisin H	345
12.1	519	C_29_H_28_O_9_	Schisantherin D	343
12.5	503	C_29_H_28_O_8_	Interiotherin A	327
12.8	533	C_27_H_34_O_11_	Phillyrin	357
16.5	311	C_13_H_12_O_9_	Caffeoyltartaric acid	149

**Table 4:** Influence of selected treatments on chlorophyll content in Spinach leaves

Treatment	Constituents (mg/100 g dw)		
	Chl a	Chl b	Chl a + b
T1	237.45 ± 3.7 d	128.31 ± 14.16 g	365.33 ± 8.67 f
T2	335.23 ± 6.45 b	123.20 ± 8.60 f	458.66 ± 7.27 b
T3	206.33 ± 7.61 e	140.74 ± 2.42 d	346.33 ± 6.15 g
T4	474.24 ± 10.41 a	312.62 ± 17.04 a	786.83 ± 20.79 a
T5	251.91 ± 18.22 c	149.80 ± 11.03 c	400.25 ± 10.83 d
T6	253.69 ± 3.05 c	100.80 ± 5.51 g	354.10 ± 22.17 f
T7	351.28 ± 13.40 c	163.45 ± 18.07 b	515.79 ± 13.15 c

*Chl a* chlorophyll A, *Chl b* chlorophyll B, *Chl a* + *b* chlorophyll A and B

## Conclusions

In the light of above findings, it is concluded that the application of bio fertilizer containing beneficial microbes displayed a stimulating effect on the soil properties and health qualities of spinach. The rhizobacterial inoculation resulted in increase of root infection by arbuscular mycorrhizal fungi. Moreover, the total phenolic compounds, antioxidant activity and chlorophyll content were also significantly enhanced by bio fertilizer treatments. These outcomes suggested that exploration of microbes display a high potential for use in the improvement of nutritious properties of fresh vegetables which could be a potential alternative to conventional approaches. However the mechanism underlying this phenomenon is not yet fully understood and will be remained for further investigations.
